# Photophysical Study on the Effect of the External Potential on NiO-Based Photocathodes

**DOI:** 10.1021/acsami.3c09566

**Published:** 2024-01-18

**Authors:** Kaijian Zhu, Lisanne M. Einhaus, Guido Mul, Annemarie Huijser

**Affiliations:** PhotoCatalytic Synthesis Group, MESA+ Institute for Nanotechnology, University of Twente, P.O. Box 217, Enschede 7500 AE, The Netherlands

**Keywords:** dye-sensitized photocathode, NiO, photodynamics, in situ spectroscopy, transient absorption

## Abstract

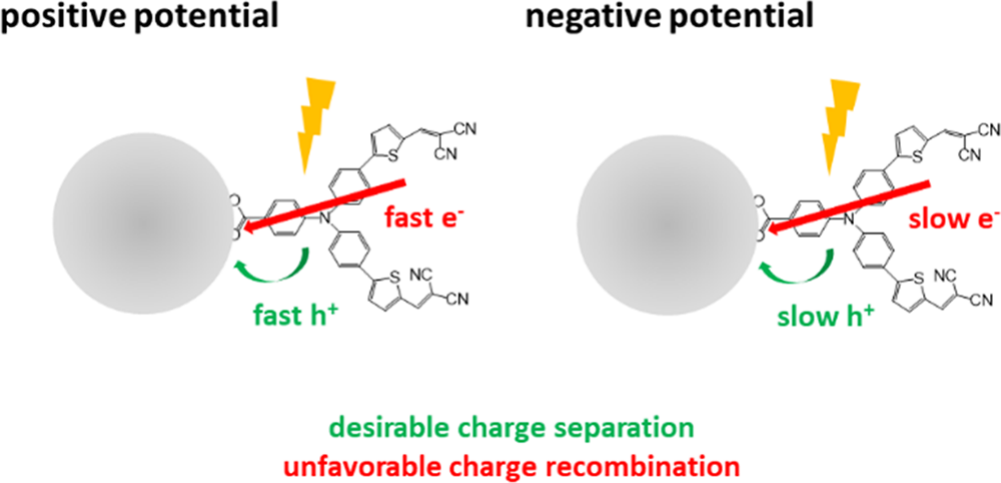

In the present study, we investigate the effects of the applied external potential on a dye-sensitized NiO photocathode by time-resolved photoluminescence and femtosecond transient absorption spectroscopy under operating conditions. Instead of the anticipated acceleration of photoinduced hole injection from dye into NiO at a more negative applied potential, we observe that both hole injection and charge recombination are slowed down. We cautiously assign this effect to a variation in OH^–^ ion concentration in the inner Helmholtz plane of the electrochemical double layer with applied potential, warranting further investigation for the realization of efficient solar fuel devices.

## Introduction

Efficient dye-sensitized photoelectrochemical (DSPEC) cells can contribute to mitigating energy and environmental challenges, by using solar energy to reduce e.g. CO_2_ into high-energy solar fuels.^1−5^ However, the overall efficiency of the tandem DSPEC cell is limited by severe charge recombination at the photocathode, which has a substantially lower performance than the photoanode.^6,7^ In a dye-sensitized photocathode, a monolayer of a molecular dye is anchored onto the surface of a p-type semiconductor and functions as a light absorber. A catalyst for proton or CO_2_ reduction can be linked to the dye, coadsorbed on the semiconductor, or even dissolved into the electrolyte.^8−13^ Light-induced charge separation by hole injection from the dye into the valence band of the semiconductor should be followed by electron transfer to the catalyst. This working principle differs from conventional photoelectrodes, based on, for example, Fe_2_O_3_ or Cu_2_O, where light absorption and charge separation take place in the same material^14,15^ In this case, the external potential (positive bias for n-type and negative bias for p-type semiconductors) facilitates light-induced charge separation and retards charge recombination in the semiconductor, leading to a higher photocurrent at more positive (photoanode)^16,17^ or more negative potential (photocathode).^18^ However, whether an applied potential has the same effect on DSPEC cells with their configuration based on light-induced charge separation at the dye–semiconductor interface rather than inside the semiconductor is unknown, although some dye-sensitized photocathodes show surprisingly low photocurrents at more negative bias potentials.^19,20^ The composition of the electrolyte may also play an important role here.

Ultrafast spectroscopy is highly useful to investigate light-induced processes in photoactive materials. Notably, most ultrafast spectroscopy studies on dye-sensitized photoelectrodes have been carried out in air or in an organic solvent instead of under operating conditions.^8,21−25^ Durrant and co-workers observed that application of a negative bias potential on a dye-sensitized TiO_2_ photoelectrode in an anhydrous electrolyte slows down photoinduced electron injection.^26,27^ Meyer and co-workers performed bias-dependent transient absorption (TA) studies on dye-sensitized nanoITO^28−30^ and nanocrystalline TiO_2,_^31^ also in organic solvent, and observed a decrease in electron injection yield at negative potentials. Changing the applied potential even enables to reverse the photoinduced electron-transfer directionality at the dye-sensitized ITO interface.^28,31^ In addition to these studies in nonaqueous environment, Lyon and Hupp observed surface protonation and deprotonation of nanocrystalline TiO_2_ in aqueous solution as a function of applied potential.^32^ Meyer and co-workers observed the incident photon-to-electron conversion efficiency of a dye-sensitized TiO_2_ photoelectrode to depend on the pH and presence of Li^+^ ions in the electrolyte.^33^

Photocathodes have been studied less than photoanodes, and primarily also in non-aqueous solvents. Papanikolas and co-workers observed that changing the external potential applied to a RuP-sensitized NiO photocathode from positive to negative increases the photoinduced hole injection efficiency from 0 to 100%.^34^ Also research by Meyer^34,35^ and others^36−38^ on dye-sensitized NiO show that a negative external potential accelerates hole injection and slows down charge recombination. However, these studies were carried out in an acetonitrile-based electrolyte, while for proton or CO_2_, reduction an aqueous electrolyte is desirable.^39,40^ Moreover, we recently observed that the working environment of a NiO-based photocathode plays an important role in both the light-induced hole injection and charge recombination dynamics.^41^ So far, the understanding of the charge separation and recombination dynamics in an aqueous electrolyte under external potential is limited.

In this work, we investigate the effect of the external bias potential on the photodynamics of a NiO-based photocathode in phosphate buffer solution (PBS) by time-resolved photoluminescence (TRPL) and femtosecond TA spectroscopy. The nanostructured NiO is functionalized with the benchmark P1 dye [4-(bis-4-(5-(2,2-dicyano-vinyl)-thiophene-2-yl)-phenyl-amino)-benzoicacid], especially designed for the functionalization of p-type semiconductors.^42,43^ We observe that both light-induced hole injection from P1 into the NiO and charge recombination strongly depend on the applied external potential and assign the trends observed to a change in ions in the inner Helmholtz plane (IHP) of the electrochemical double layer. Our work demonstrates the important role of bias-dependent ion adsorption in the electrochemical double layer on the interface photodynamics during operation.

## Results and Discussion

**Figure 1:**
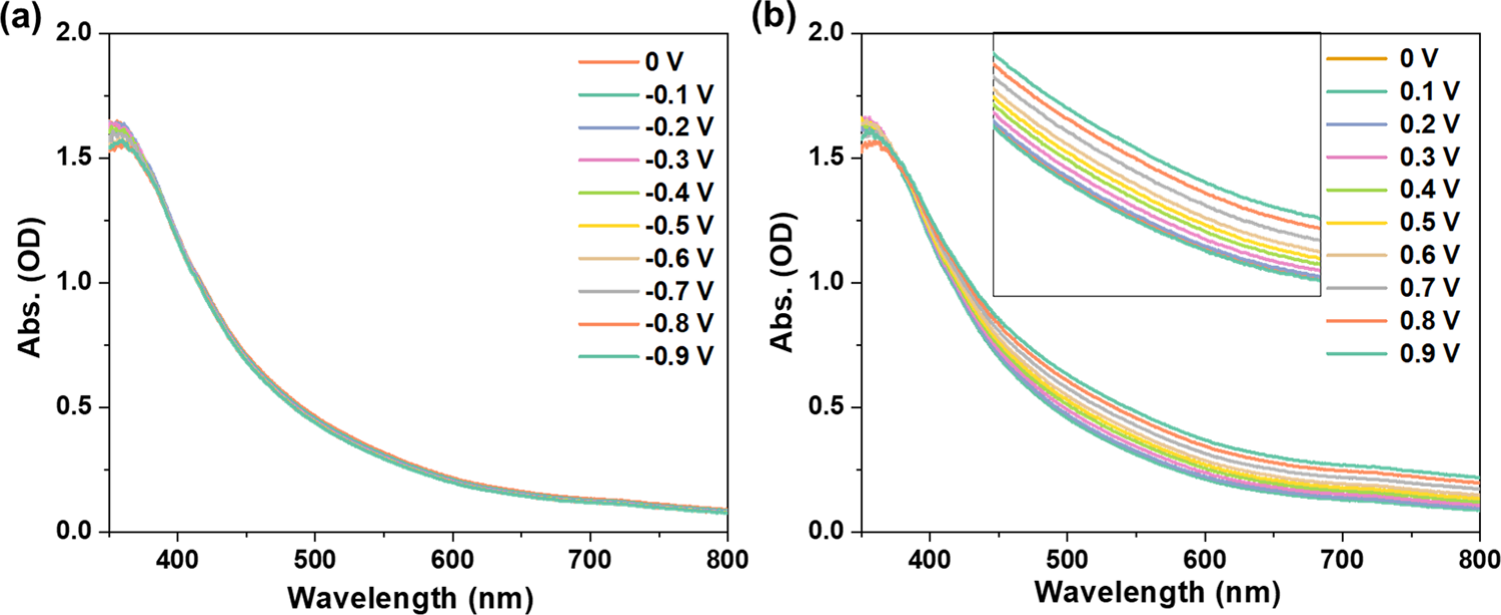
UV–vis absorbance spectra of NiO in PBS electrolyte (0.1 M, pH 7) under various negative (a) and positive (b) external potentials vs Ag/AgCl.

The scanning electron micrographs and X-ray diffraction patterns of the oxidized Ni on fluorine-doped tin oxide (FTO) substrates are shown in Figures S3 and S4, demonstrating NiO with a highly porous layer structure extending out of the FTO surface by approximately 2 μm. Figure 1a shows the UV–vis absorbance spectra of the NiO film in PBS (pH = 7, the commonly used electrolyte^1,11^) at various applied negative potentials from 0 to −0.9 V. The spectra are very similar, indicating that reduction of Ni^2+^ to Ni is minor or negligible in this potential range. However, with a potential change from 0 to +0.9 V, the visible absorbance of the NiO film increases substantially (Figure 1b), initiating at ∼0.3 V and becoming even more pronounced from potentials around 0.7 V. It is widely accepted that NiO may show a gray or black color due to a large amount of defects (Ni^3+^).^44^ However, the oxidation potential of Ni^2+^ to Ni^3+^ is around 1.4 V vs RHE (reversible hydrogen electrode, i.e. ∼0.7 V vs Ag/AgCl, see Figure S5).^45−47^ Therefore, the increase in visible light absorption <0.7 V in Figure 1b is not due to electrochemical oxidation of bulk NiO, which is further confirmed by the XPS results in Figure S4. Therefore, we believe that surface-related hydroxylation phenomena forming different phases of Ni–OH in the IHP,^48−50^ possibly combined with (un)filling of trap states with changing the applied bias potential,^51^ are responsible for the observed changes in UV–vis spectra. These processes could be correlated: Boschloo and co-workers reported that electro-adsorbed cations act as trap states for electrons in dye-sensitized TiO_2_.^52^

The quantity of OH^–^ ions in the IHP, adsorbed on the NiO surface, will increase with a more positive potential, while a negative potential will promote H^+^ adsorption. Furthermore, the extraction/intercalation of H^+^ and OH^–^ ions from/into the NiO films can lead to electrochromic phenomena.^53,54^ Therefore, we assign the changes in the UV–vis spectra with applied potential to compositional surface intercalation associated with the IHP.

**Figure 2:**
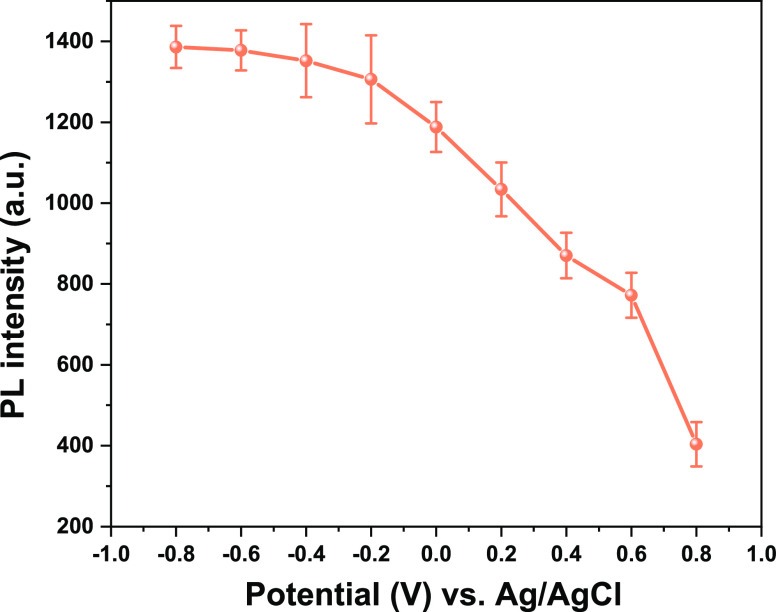
Photoluminescence intensity at 670 nm of NiO/P1 in PBS electrolyte (0.1 M, pH 7) following excitation at 532 nm as a function of the applied external potential. The data points are the maximum intensities measured by TRPL (Figure S7), corrected for bias-dependent filtering effects by the NiO at 532 nm excitation and 670 nm emission. The observed trend with changing the potential is reversible, showing that dye leaching is negligible.

To elucidate the photoinduced dynamics of NiO/P1 in the PBS electrolyte under various external bias potentials, the spectrotemporal photoluminescence (PL) behavior was measured by streak camera detection (Figure S7). As bare NiO does not show any PL in this wavelength range following excitation at 532 nm, the PL signal in Figure S7 primarily originates from the excited P1 dye (P1*). On an insulating ZrO_2_ support, the PL lifetime of P1* equals ca. 250 ps.^41^ Light-induced hole injection from P1* into NiO is known to occur in a few hundred fs to several ps.^55^ Although, as a result, the PL decays of P1 on NiO are within the instrumental response time of the streak camera, the PL intensity as a function of applied potential shown in Figure 2 is indicative of the hole injection rate from P1* into the NiO causing PL quenching. A positive potential leads to strong PL quenching, while a negative potential has less effect. As other quenching mechanisms may also play a role and be bias-dependent, we quantified the hole injection time constants at various bias potentials by femtosecond TA experiments discussed in detail below, which confirm that a positive potential accelerates photoinduced hole injection. This faster hole injection can be understood by the dual function of surface-adsorbed OH^–^ in the IHP, which we recently observed to promote both hole injection and recombination,^41^ with the concentration increasing with more positive potential. Considering the dimensions of the P1 dye, we assume band bending in NiO/PBS and NiO/P1/PBS to be similar. Band bending is usually small in nanoparticle films, because it decreases with the size of the semiconductor particle.^56^ NiO shows a very weak PL signal at 0 to −0.8 V following excitation at 267 nm, while the PL is more intense at positive potentials (Figure S8). According to the dead layer model, the thickness of the dead layer increases with more band bending (Figure S9), leading to a lower PL intensity.^56,57^ Hence, the higher PL intensity of NiO at a more positive potential indicates less band bending. Therefore, a significant effect of band bending in the NiO on the trend shown in Figure 2 can be excluded from the PL data obtained for NiO in PBS using 267 nm excitation. As a more positive potential implies a decrease in energy level difference between the valence band of the NiO and the HOMO of the P1 dye, this cannot explain the faster hole injection indicated by the trend in Figure 2. The opposite dependency of the PL intensity on the applied bias potential observed for ZrO_2_/P1 following excitation at 532 nm (Figure S10) compared to NiO/P1 (Figure 2) suggests that a Stark effect^58^ and changes in the solvation shell of the P1 dye molecules do not play significant roles in the bias-dependent data of NiO/P1.

**Figure 3:**
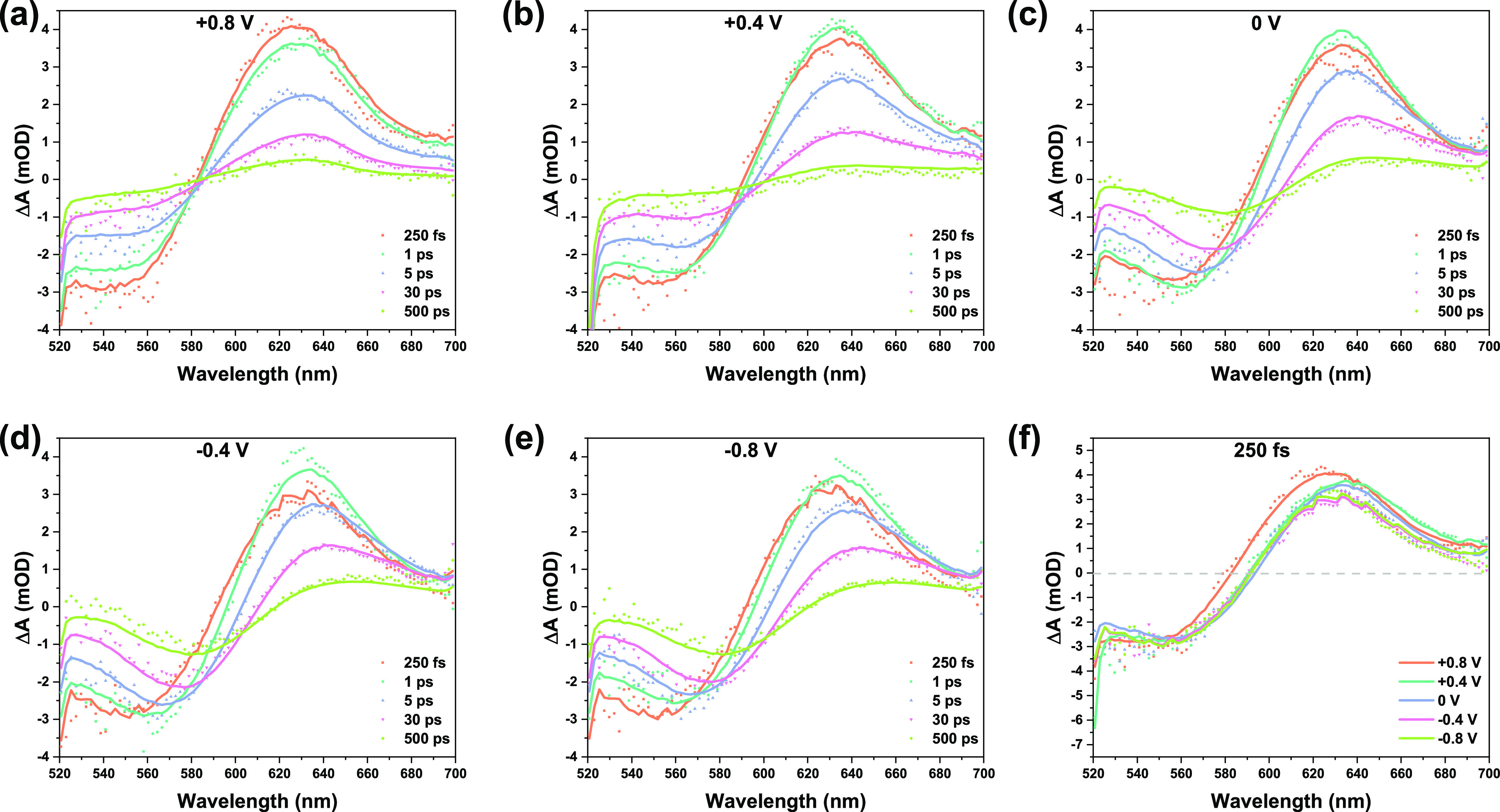
Transient absorption spectra at different time delays after excitation at 500 nm of NiO/P1 in PBS electrolyte (0.1 M, pH 7) under various external potentials (a–e) and the spectra at 250 fs (f). The solid lines indicate results from target analysis.

**Figure 4:**
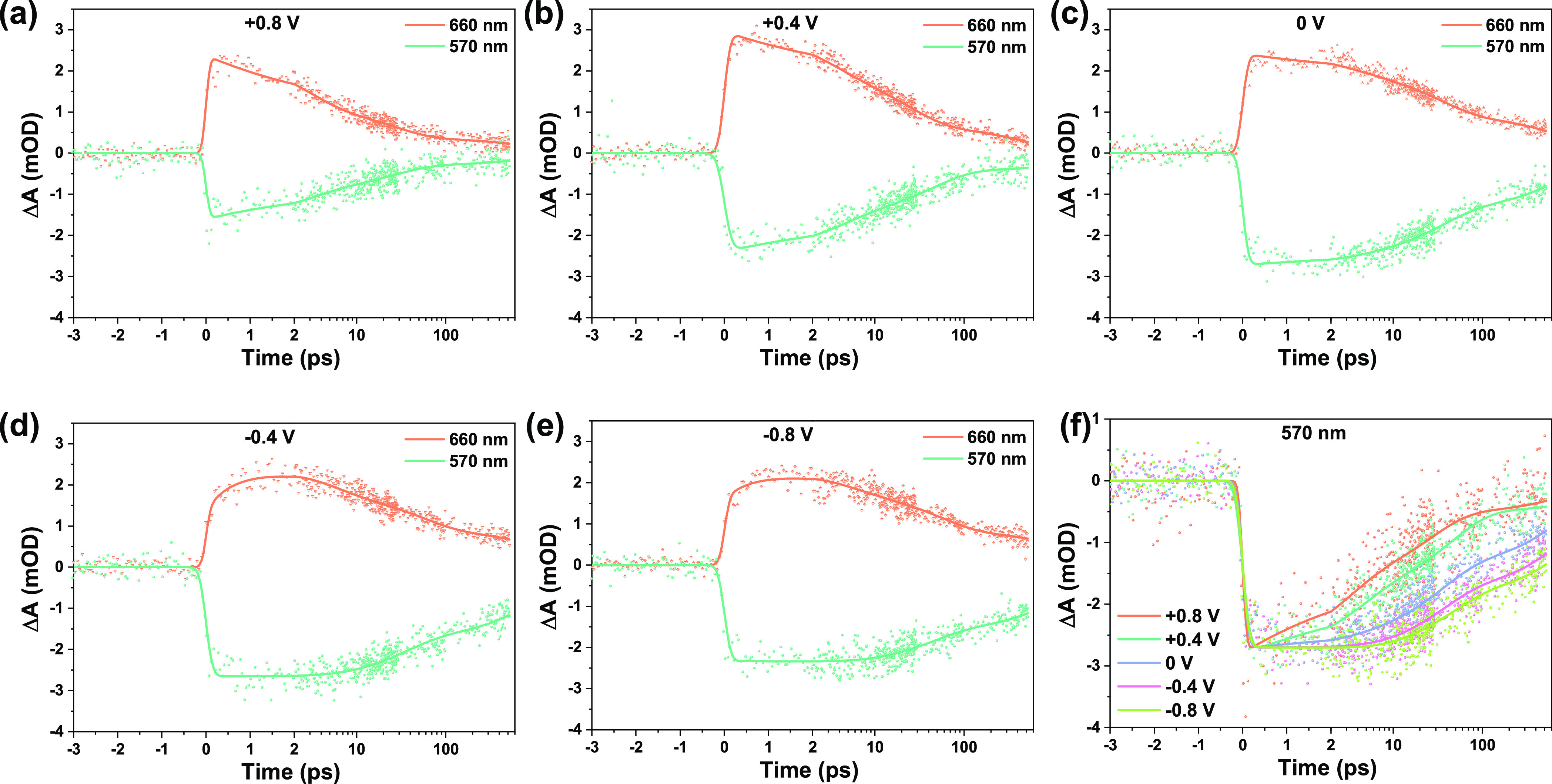
Transient absorption kinetic traces at 570 and 660 nm after excitation at 500 nm of NiO/P1 in PBS electrolyte (0.1 M, pH 7) under various external potentials vs Ag/AgCl (a–e). In (f), the kinetic traces at 570 nm at various applied potentials are shown. The solid lines indicate results from target analysis.

Femtosecond TA studies were performed to further investigate the role of the external potential in the interfacial photodynamics of NiO/P1 in the PBS electrolyte. Figure 3a–e shows the TA spectra at various applied potentials. These data have been recorded on the same NiO/P1 sample to avoid any potential effect of sample-to-sample variations. The broad negative signal is due to the photoinduced ground state bleach (GSB) of the excited P1 dye.^41,55,59^ P1* is known to have a strong and broad positive absorbance around 550–560 nm.^41,55,59^ Due to hole injection from P1* into NiO, the P1* signal decreases and the characteristic absorbance around 610 nm of P1^·–^ arises, causing a red-shift in the spectrum with time. Hole injection from P1* into NiO is typically a biphasic process, with the fastest component within the TA Instrumental response time (IRT, 100–150 fs).^55^ The early time spectra can hence be used to assess whether the applied potential affects the extent of ultrafast hole injection within the IRT. However, the spectrum at 250 fs at +0.8 V is blue-shifted and broader than the spectra at less positive potentials (Figure 3f), which seems to be in contradiction with the faster hole injection indicated by stronger PL quenching (Figure 2). According to the PL results (Figure 2) and TA kinetic traces (Figure 4), the blue shift in Figure 3f is not caused by slower hole injection. The difference in the absorption spectrum between Ni^4+^ and Ni^3+^ is the likely reason. At potentials below 0.6 V, hole injection from P1* into NiO likely leads to oxidation of Ni^2+^ into Ni^3+^, while at +0.8 V, the same process results in Ni^3+^/ Ni^4+^ oxidation, with Ni^4+^ showing a visible absorption above 560 nm.^60^

Figure 4a–e compares the TA kinetic traces at 570 and 660 nm, at different potentials. The transient signal at 570 nm is predominantly due to P1* (positive signal), GSB (negative signal), and P1^·–^ formed due to hole injection (positive signal), while the signal at 660 nm is primarily due to P1^·–^.^41,55,59^ When NiO/P1 is exposed to an aqueous solution or to air with a high relative humidity, most of the hole injection occurs ultrafast and is finished within the IRT (100–150 fs); only a minor part of the hole injection occurs slower (1–2 ps).^41^ The strong PL quenching especially at +0.8 V (Figure 2) already indicates that hole injection from P1* into NiO occurs the fastest at these conditions and becomes gradually slower on moving toward a negative applied potential, which is supported by the TA kinetic traces at 660 nm. At positive potentials, the TA signal fully develops within the IRT, while at negative applied potentials, a minor further ∼1 ps rise is observed indicating a slower hole injection component. Comparison of the transient signals at 570 nm at different potentials (Figure 4f) shows that charge recombination becomes slower with a more negative potential. Retardation of charge recombination at negative potential was reported earlier and explained by filling of intragap trap states.^34,61^ However, in that study performed in acetonitrile, hole injection is also accelerated by a more negative potential,^34^ in contrast to the trend shown in Figures 2 and 4. A possible reason for this contrast is the strong dependency of the NiO surface termination on the working environment. Considering the dual role of surface OH^–^ we unraveled recently, accelerating both hole injection and charge recombination,^41^ the dependency of the photodynamics on the external potential observed here is likely due to a change in ions (H^+^ or OH^–^) in the IHP. The quantity of surface OH^–^ ions is the highest at positive potential, resulting in both fast photoinduced hole injection and charge recombination. This dual role of surface OH^–^ gradually decreases when moving to negative potentials. To provide additional evidence of the effect of H^+^ and OH^–^, we also carried out a new experiment. We measured the TA on the same NiO/P1 sample in two different pH values (pH 4, i.e. a high H^+^ concentration and pH 10, i.e. a high OH^–^ concentration). The kinetic traces at 570 nm are shown in Figure S11. In a pH 10 solution, the trace is comparable to those at +0.8 and +0.4 V (fast few ps decay, Figure 4a,b), while in pH 4 it is comparable with the traces at 0 to −0.8 V (no few ps change in signal, Figure 4d,e). The difference between pH 10 and pH 4, for the same sample, strongly indicates that the different concentrations of OH^–^ and H^+^ are responsible for the changes in interface photodynamics with applied bias potential. We cautiously assign the small difference between −0.4 and −0.8 V to a saturation in the quantity of surface-absorbed H^+^ in the IHP.

**Figure 5:**
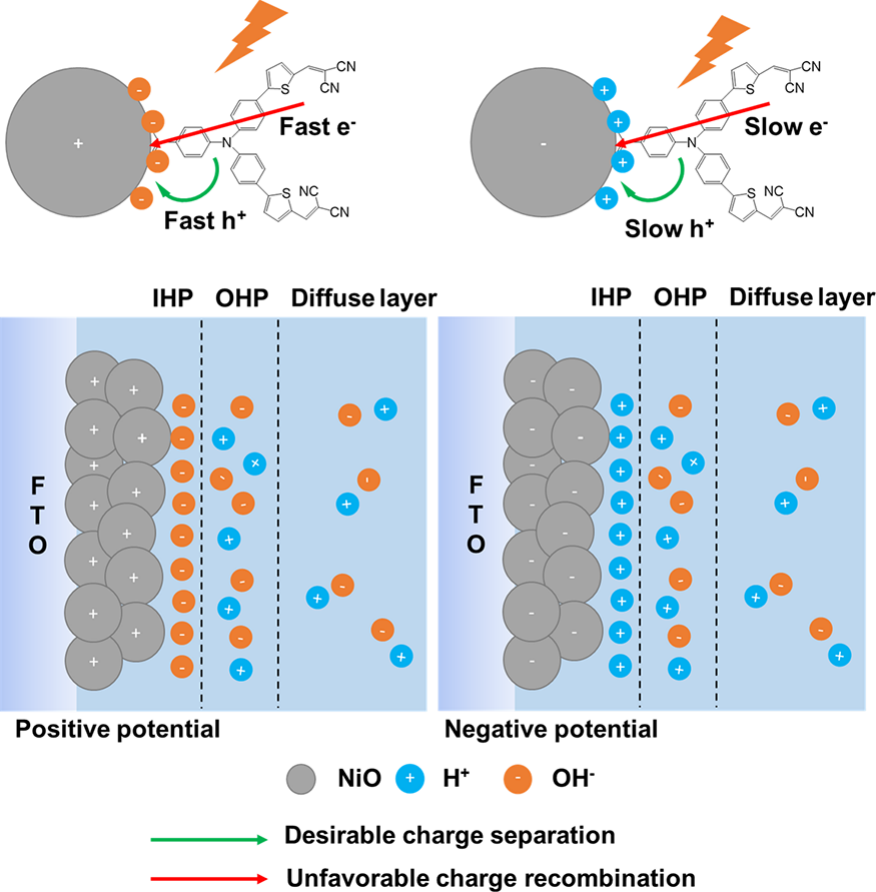
Proposed model to explain the effect of the positive and negative external potentials on the NiO/P1 interface photodynamics. IHP: inner Helmholtz plane; OHP: outer Helmholtz plane.

Figure 5 presents a possible model that can explain the effect of the different external potentials on the interface photodynamics of NiO/P1 in PBS observed here. Band bending will be small for these small NiO nanoparticles.^56^ The TRPL data discussed above confirm that effects of band bending on the observed potential dependency are insignificant. We therefore focus on the electrode–electrolyte interface, at which two types of electrochemical processes can occur. One is the Faradaic process, i.e., the oxidation or reduction reaction. The second type is the non-Faradaic process, in which surface adsorption and desorption occur and the structure of the electrode/electrolyte interface changes with applied bias potential and electrolyte composition.^62^ For NiO, the Faradaic process leading to Ni^2+^/Ni^3+^ oxidation predominantly occurs at ∼1.4 V vs RHE, i.e. ∼0.7 V vs Ag/AgCl^45−47^ (see also Figure S3 for a cyclic voltammogram of NiO in PBS), implying that at lower potential, cation (H^+^) and anion (OH^–^) adsorption onto the NiO surface and desorption into the PBS are the dominant processes. Other ions like K^+^, PO_4_^2–^, or HPO_4_^–^ might also play a role, but we observed earlier that their effect on the photodynamics is insignificant compared to OH^–^ (H^+^),^41^ which we observed to promote (slow down) both photoinduced hole injection and charge recombination. A significant effect of dissolved O_2_ or CO_2_ is also unlikely, as we purged the solution with N_2_ prior to the experiments, and we observed similar photodynamics without and with prior N_2_ purging.^41^ The present work demonstrates that the interfacial photodynamics change with the applied external potential before oxidation of Ni^2+^ into Ni^3+^ occurs. Therefore, we propose that the non-Faradaic process, i.e. ion adsorption and desorption onto the NiO surface, or ion adsorption causing surface density state changes, plays an essential role as a relay in the light-induced charge transfer and recombination processes. Our interpretation is in line with earlier work on effects of surface-adsorbed ions on dye-sensitized TiO_2_ photoelectrodes.^32,33^ This effect has not yet gained much attention due to the lack of ultrafast spectroscopy studies under in situ conditions. The positively charged NiO attracts OH^–^ ions into the IHP, which become adsorbed onto the NiO surface and as a result accelerate light-induced hole injection and charge recombination. In contrast, negatively charged NiO favors H^+^ adsorption, slowing both hole injection and charge recombination but with less dependency on the applied potential compared to positive potential.

These effects have been quantified by target analysis to account for the overlap in TA signals and include bias-dependent oxidation states in the NiO using the open source program Glotaran^63^ and the photophysical models shown in Figure S12. The species associated spectra from target analysis and a detailed explanation are given in the Supporting Information and Figure S13. Table 1 presents the obtained lifetimes with τ_1_ hole injection from P1* into the IHP and τ_2_ hole transfer from the IHP into the NiO. Charge recombination is assumed to occur either between P1^·–^ and holes localized at the NiO surface (τ_3_) or in the NiO bulk (τ_4_). The lifetimes are the longest at −0.8 V and gradually become shorter with more positive potential. In summary, our results show that the adsorbed ions play a more important role in the photoinduced interface charge-transfer dynamics than the band bending in the dye-sensitized NiO photocathode induced by the applied potential. This can explain the different results in this work (negative potential leads to slower photoinduced hole injection in aqueous solution) and in the literature (negative potential promotes hole injection in acetonitrile).^34^ Our work highlights the key role of ions in the IHP and at the photocathode surface, controlled by the external bias potential, in the realization of efficient solar fuel devices.

**Table 1:** Time Constants from Target Analysis of NiO/P1 in PBS Electrolyte (0.1 M, pH = 7) under Various External Potentials vs Ag/AgCl

potential (V)	τ_1_ (fs)	τ_2_ (ps)	τ_3_ (ps)	τ_4_ (ps)
+0.8	IRT	3.2 ± 0.1	48.2 ± 1.0	∞
+0.4	IRT	4.1 ± 0.1	51.8 ± 1.1	∞
0	IRT	4.7 ± 0.1	63.2 ± 0.8	∞
–0.4	776.2 ± 25	6.5 ± 0.1	79.0 ± 1.3	∞
–0.8	1115 ± 38	7.2 ± 0.1	78.3 ± 1.4	∞

## Conclusions

This in situ TRPL and femtosecond TA spectroscopy study uncovers a major effect of the applied external potential on the interface photodynamics of NiO-based and presumably other p-type metal oxide semiconductor-based photocathodes in aqueous electrolyte for applications including solar water splitting and CO_2_ reduction. We show that the main effects of the external potential possibly arise from changes in surface ion adsorption in the IHP, playing a role as a nondirectional charge transfer relay. This study suggests that regulating water dissociation through the use of ion additives^64^ may be a simple way to improve the performance in PEC.

## Supporting Information Available

Three electrode cell used in this work; UV-Vis absorbance spectra of the P1 dye in ethanol, on NiO and on ZrO_2_; surface and cross-sectional SEM images of NiO on FTO; XRD patterns of NiO on FTO including the assignments of the diffraction lines^2^ and the bare FTO substrate; cyclic voltammogram of NiO in PBS (pH=7) recorded with a 5 mV/s scan rate; Ni 2p XPS spectra of the NiO film; time-resolved photoluminescence decay profiles of NiO/P1 in PBS electrolyte; band bending in a p-type material under different potentials; time-resolved photoluminescence decay profiles of ZrO_2_/P1 in PBS electrolyte; transient absorption kinetic traces at 570 nm after excitation at 500 nm of same NiO/P1 sample in the solution with different pH; photophysical model used to describe the femtosecond transient absorption data; species associated spectra obtained from target analysis of the TA data of NiO/P1 in PBS electrolyte; and time constants from target analysis of NiO/P1 in PBS electrolyte (PDF)
