# Supplementary material for: Photophysical Study on the Effect of the External Potential on NiO-Based Photocathodes
Source: ACS Appl Mater Interfaces. 2024 Jan 18;16(4):5217–24. doi: 10.1021/acsami.3c09566 (PMC10835655; doi:10.1021/acsami.3c09566)
Supplement: Supplementary file 1 — am3c09566_si_001.pdf [file am3c09566_si_001.pdf]

Supporting information

Photophysical Study on the Effect of the External Potential on NiO-Based Photocathodes

Kaijian Zhu,[†] Lisanne M. Einhaus,[†] Guido Mul,[†] Annemarie Huijser *[†]

[†]PhotoCatalytic Synthesis Group, MESA+ Institute for Nanotechnology, University of Twente, P.O. Box 217, 7500 AE, Enschede, the Netherlands

Experimental Section

Sample Preparation

The NiO films were prepared by a chemical bath deposition method, followed by annealing. First, the fluorine-doped tin oxide (FTO, Sigma-Aldrich) substrates were cleaned by acetone, isopropanol, and ethanol. The cleaned FTO plates were treated in a UV-ozone photoreactor (UVP PR-100) for 30 min., after they were cut in 20 × 20 mm pieces. Two sides of the plates were covered along the full length with adhesive tape (Scotch® MagicTM tape 8-1933R8), and ZrO_2 nanoparticle paste (Solaronix, ZT/SP Ref 46411) was applied on the top of the plate with a glass rod. The paste was then smeared from top to bottom using the edge of a glass plate, which was held at <30° angle. This process was repeated until a homogeneous layer of the paste was obtained. The tape was removed and the plates were thermally annealed at 500 °C for 30 min. (with a temperature ramp of 20 °C/min.). The plates were taken out of the furnace immediately after the program finished.

The NiO films were prepared by a chemical bath deposition method, followed by annealing. The 0.075 M $Ni(NO_3)_2$•6 H_2O (Sigma-Aldrich, 99.999%), 0.2 M urea (Sigma-Aldrich, >99%) and 0.75 M ethanolamine (Sigma-Aldrich, >99%) precursors were sequentially dissolved into Milli-Q water. Then FTO substrates were put into the solution, and films were grown on the FTO at 90 °C for 3 hours and

washed by Milli-Q water. Finally, the NiO films were obtained by annealing in air at 450 °C for 1 hour. The films were immersed into 0.3 mM P1 dye solution (4-(bis-4-[5-(2,2-dicyano-vinyl)-thiophene-2-yl]-phenyl-amino)-benzoic acid, Dyenamo, Sweden) in ethanol (Supelco, >99.9 %) in the dark for ca. 16 hours and washed by ethanol, resulting in a monolayer coverage with chemisorbed dye molecules.

Characterization

All the in-situ experiments were carried out in a three-electrode quartz cell (10x10 mm, Hellma, 101 – Macro cells, Figure S1) with the NiO/P1 film as the working electrode, a Ag/AgCl reference electrode and a Pt wire as the counter electrode. The electrolyte used was a 0.1 M phosphate buffer solution (PBS) with a pH value around 7. Before each experiment, the electrolyte was degassed by N_2 for more than 30 min. The UV-Vis absorbance spectra of the films were recorded using a ThermoSci EVO600 spectrometer. The valence states of the NiO under different applied potentials were analyzed immediately after applying the potential for 5 min. by X-ray Photoelectron Spectroscopy (PHI Quantera SXM). The 5 min. treatment should be sufficient to achieve a homogeneous oxidation profile across the entire NiO layer. Electrochemically oxidized Ni^{3+} can be stored even without electrolyte due to its capacitive properties[1] and should therefore be detectable by ex-situ XPS. The nanomorphology of the layers was studied by a Zeiss MERLIN HR-SEM.

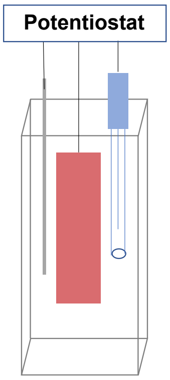

Figure S1. The three electrode cell used in this work.

Time-resolved photoluminescence spectroscopy

The setup used for time-resolved photoluminescence experiments was described in detail in our previous work.[2] Briefly, a Fianium laser (FP-532-1-s, center wavelength 532 nm, pulse duration of 300 fs, 80.37

MHz repetition rate) was used as the light source. For experiments with UV excitation (267 nm), the Fianium output was focused into a 3 mm β-BaB_2O_4 crystal (Newlight Photonics). The residual 532 nm output was removed by using three dichroic mirrors (Thorlabs, MBI-K04) and a FGUV11-UV filter (Thorlabs). The $\lambda_{exc.}$ = 267 nm and $\lambda_{exc.}$ = 532 nm experiments were performed using a power of 27 μW and 8.2 μW, respectively. The sample was kept in a quartz cuvette (Hellma, 10 mm optical path length) as the working electrode with a 0.1 M phosphate buffer solution (PBS, pH=7) as the electrolyte, a Pt counter electrode and an Ag/AgCl reference electrode. The applied potential was controlled by a Emstat3 potentiostat (PalmSens). The spectral calibration was checked and adapted if necessary using a Hg/Ar calibration lamp (Oriel, LSP035). The spectral sensitivity of the photoluminescence spectra was corrected by the equations below, which were determined by measuring the spectrum of a black body lamp (Ocean Optics, HL-2000) with its calibrated spectrum:

$$Real\ PL\ spectrum = \frac{measured\ PL\ spectrum}{Y}$$

$$Y = 110 - 0.0012\ (wavelength\ in\ nm - 600)^2$$

Femtosecond transient absorption spectroscopy (fs TA)

The setup used for femtosecond transient absorption experiments was described in detail in our previous work.[2,3] To avoid a potential (verified to be minor) role of sample variation, all comparative experiments were performed on the same NiO/P1 sample.

Supplementary results

The UV-Vis absorbance spectra of P1 in ethanol, deposited onto NiO or ZrO_2 are shown in Fig. S2. Due to electronic coupling between dye and semiconductor, the UV-vis spectra of the dye on the metal oxide show a broadening and red-shift in absorbance compared to the dye in solution, similar as in the literature[4]. A difference in electronic coupling between the P1 dye and ZrO_2 and NiO can also explain the small difference between the spectra of P1 on ZrO_2 and NiO.

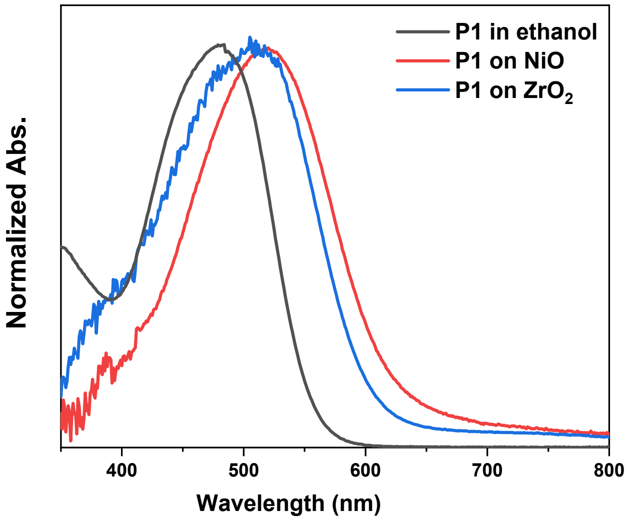

Figure S2. UV-Vis absorbance spectra of the P1 dye in ethanol, on NiO and on ZrO_2, corrected for the signal of the substrate, if used.

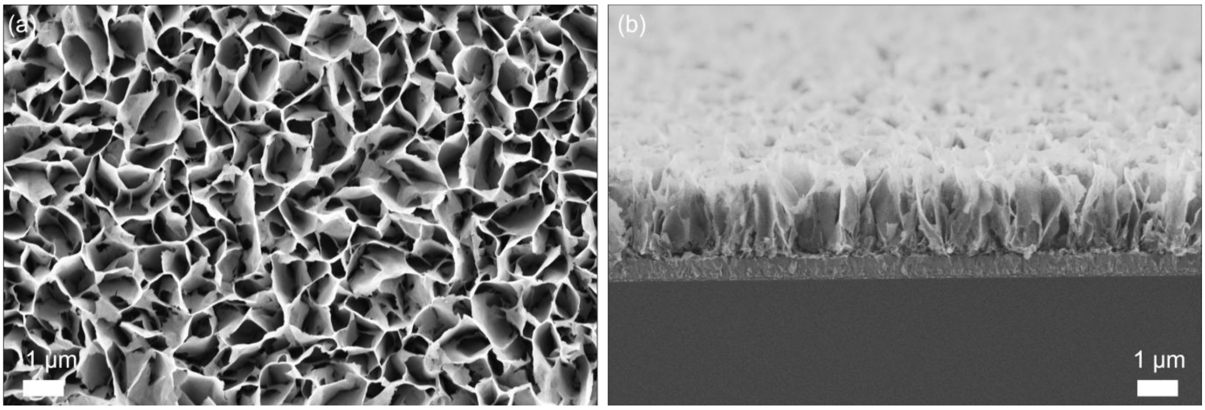

Figure S3. Surface (a) and cross-sectional (b) SEM images of NiO on FTO.

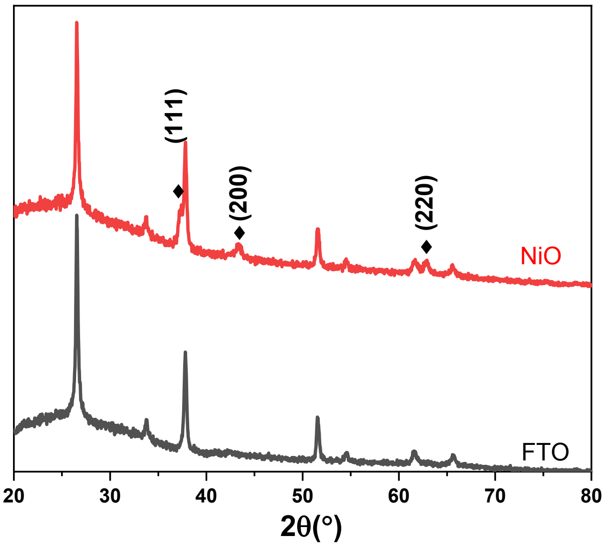

Figure S4. XRD patterns of NiO on FTO including the assignments of the diffraction lines[2], and the bare FTO substrate.

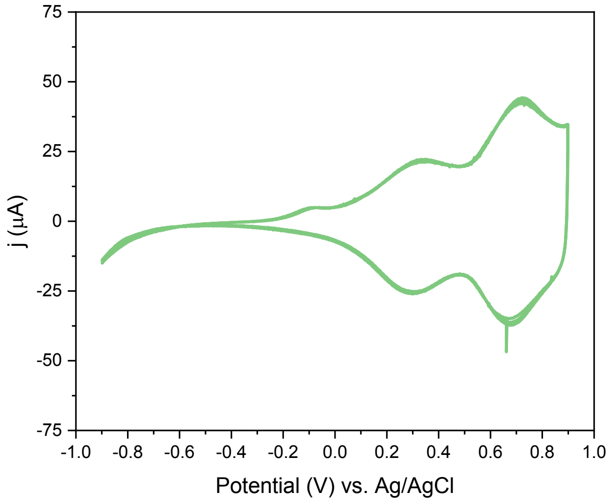

Figure S5. Cyclic voltammogram of NiO in PBS (pH=7) recorded with a 5 mV/s scan rate.

Figure S3 shows the surface and cross-sectional SEM images of NiO on FTO, and Figure S4 the XRD patterns of NiO on FTO, including the assignment of the diffraction lines, and the FTO substrate. Figure S5 shows the cyclic voltammogram of NiO in PBS recorded with 5 mV/s scan rate. The peak at around 0.7 V can be assigned to the Ni^{2+} to Ni^{3+} oxidation[5-7] and the other signals to the capacitive current. Reduction of Ni^{2+} to Ni is minor or negligible in this voltage range.

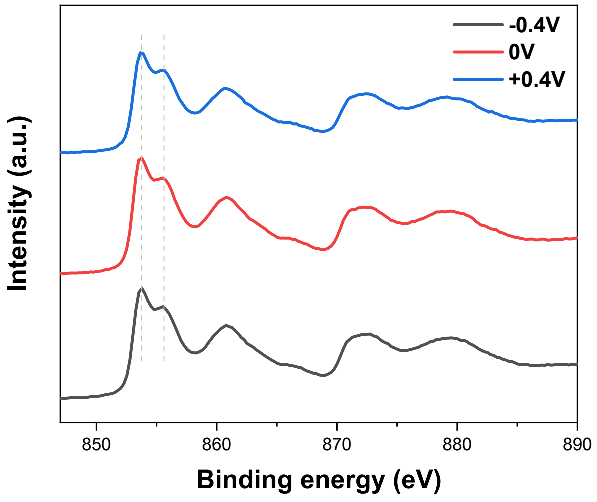

Figure S6. Ni 2p XPS spectra of the NiO film immediately after applying the indicated external potential in pH = 7 PBS electrolyte.

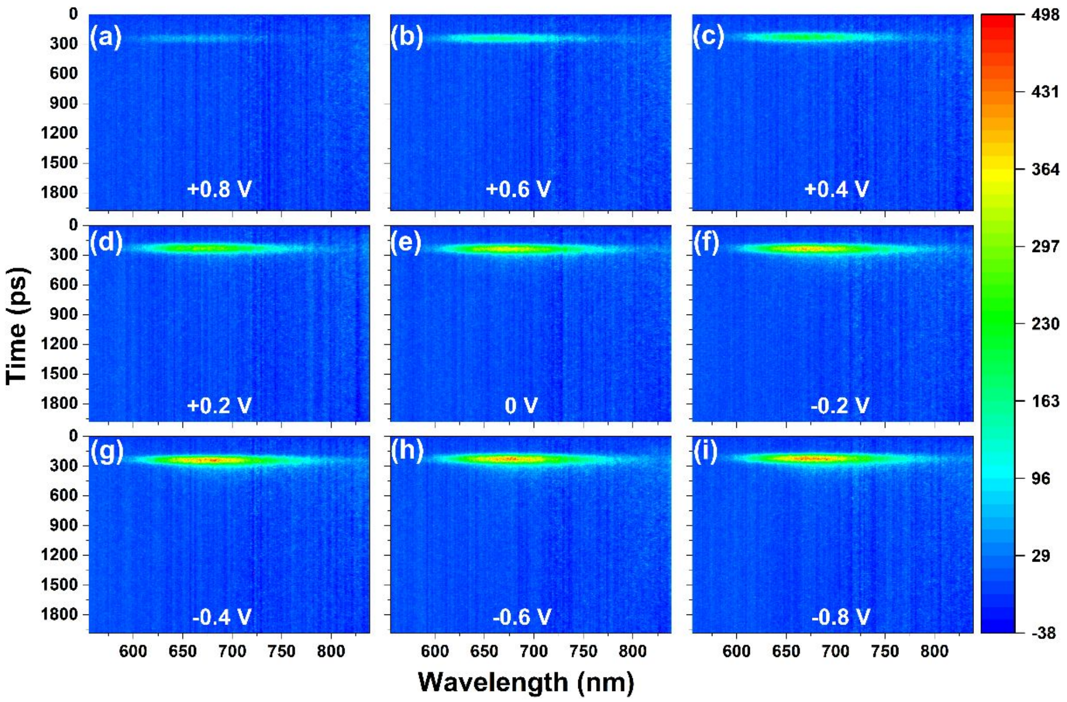

Figure S7. Time-resolved photoluminescence decay profiles of NiO/P1 in PBS electrolyte (pH = 7) at various external potentials following excitation at 532 nm. The potential is vs. Ag/AgCl.

Figure S6 shows the Ni 2p XPS spectra of the NiO film immediately after applying different external potentials in pH = 7 PBS electrolyte. Figure S5 shows the photoluminescence decay profiles of NiO/P1 in PBS electrolyte (pH = 7) at various external potentials following excitation at 532 nm. As NiO does not show any emission in this wavelength range after 532 nm excitation, the signal in Figure S5 primarily originates from the excited P1 dye (P1*), which decays quickly due to ultrafast hole injection into the NiO. Although as a result the photoluminescence decays of P1 on NiO are within the instrumental response time of the streak camera, the photoluminescence intensity as function of applied potential shown in Figure S7 is indicative of the hole injection rate from P1* into the NiO causing PL quenching.

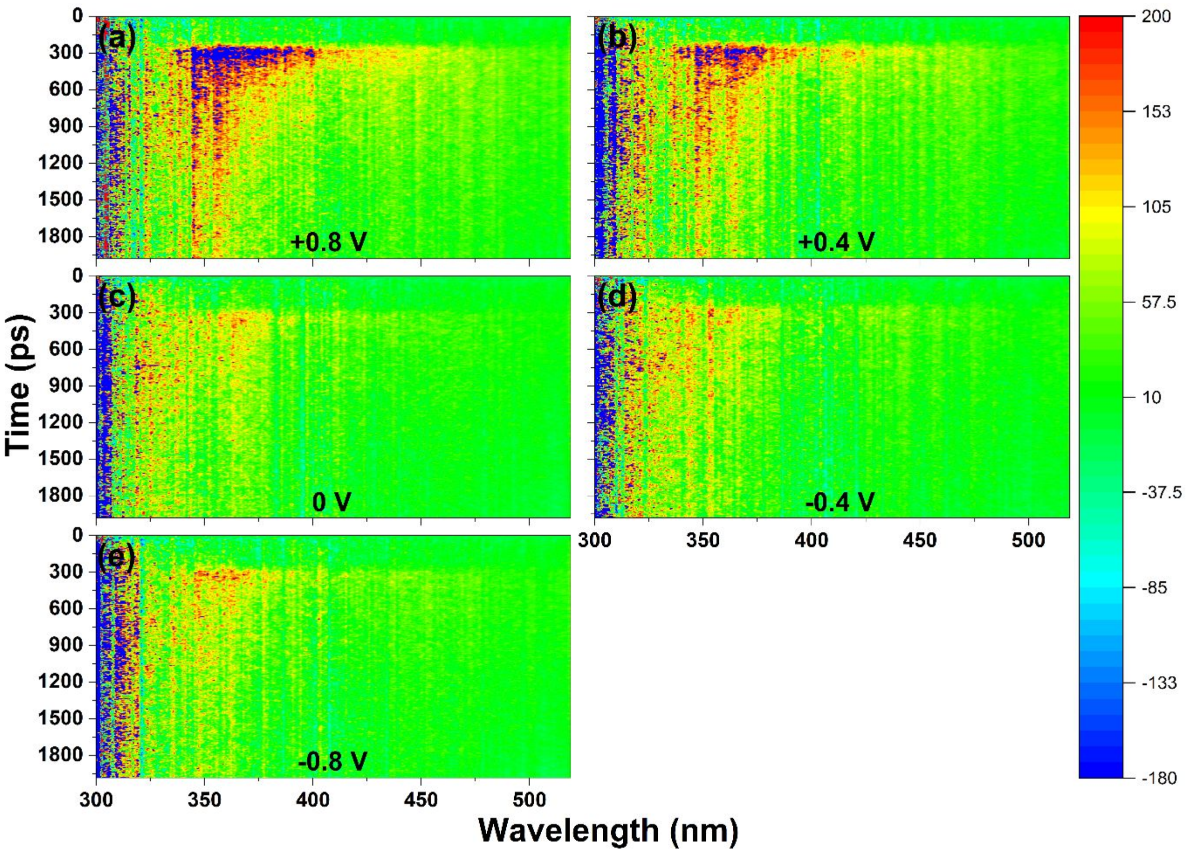

Figure S8. Time-resolved photoluminescence decay profiles of NiO in PBS electrolyte (pH=7) under various external potentials following excitation at 267 nm.

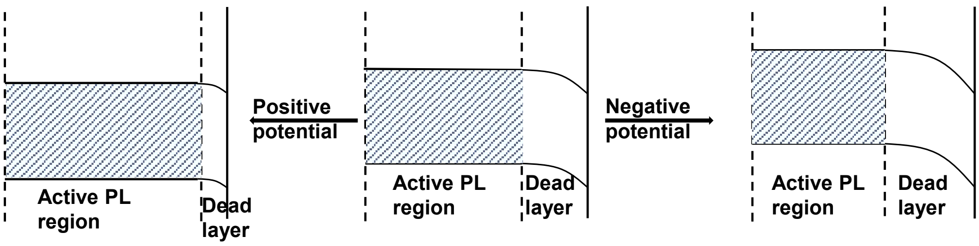

Figure S9. Band bending in a p-type material under different potentials. For simplicity the illustration neglects possible effects of Fermi level pinning.

Figure S8 shows the photoluminescence decays of NiO films in PBS following excitation at 267 nm at various applied potentials. The spectra at +0.8 V and +0.4 V show a higher photoluminescence intensity,

which can be explained by less band bending (see schematic diagram in Figure S9). According to the dead layer model, the photoluminescence intensity relates to band bending.[8,9] With more band bending, the dead layer is thicker, which results in a lower photoluminescence intensity originating from the part of the layer without band bending. NiO is a material with an indirect band gap and a defect-rich surface, resulting in a very weak photoluminescence signal. In PBS, the additional band bending caused by the NiO/electrolyte interface leads to quenching of most of the PL signal, both at 0 V and at negative potentials.

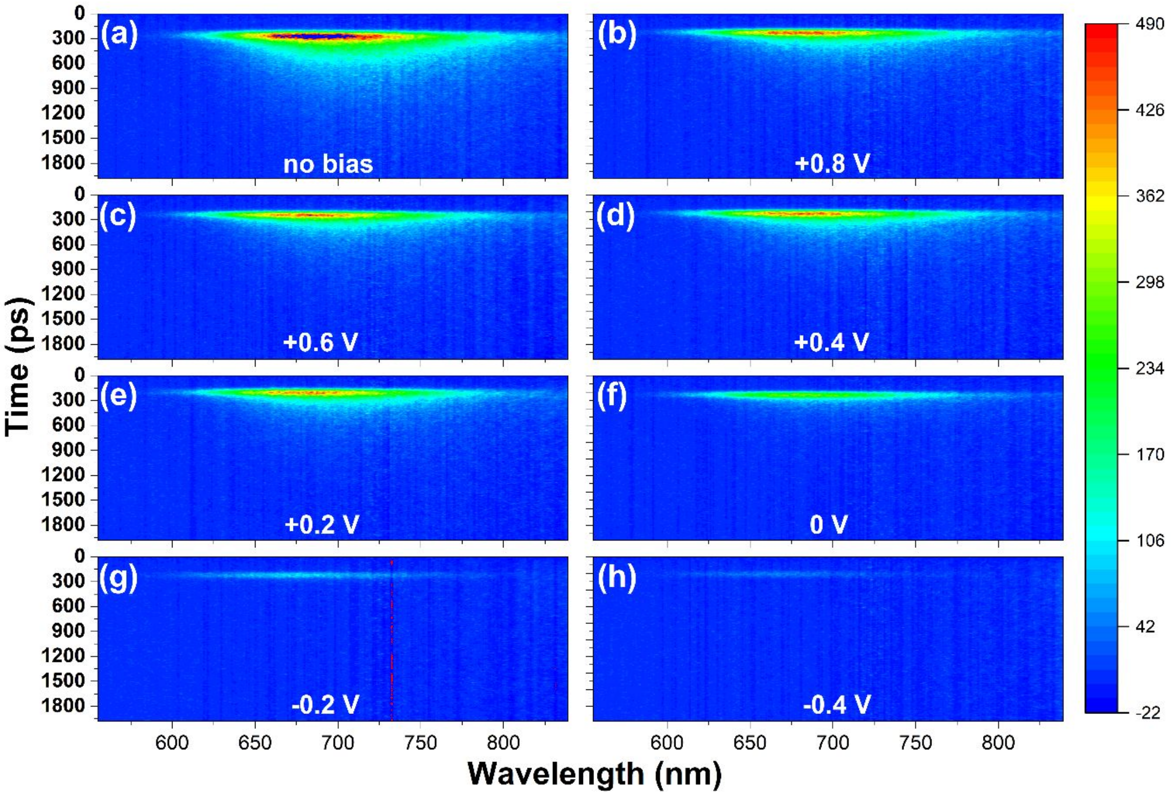

Figure S10. Time-resolved photoluminescence decay profiles of ZrO_2/P1 in PBS electrolyte (pH=7) under various external potentials after excitation at 532 nm.

Figure S10 shows the PL decay profile of ZrO_2/P1 in PBS electrolyte under various external potentials following excitation at 532 nm. Recent work by Tian *et al.*[10] on ZrO_2 sensitized with the PB6 dye shows that potential induced changes in UV-VIS spectra are predominantly due to the dye. It is obvious that both the PL intensity and lifetime of P1 on ZrO_2 are quenched by the external potential, although the effects appear to be stronger at negative potential than at positive potential. Note that this trend deviates

significantly from that observed for NiO/P1 (Figure 2). The PL quenching observed for P1 on the insulating ZrO_2 support may result from changes in the solvation shell and alterations in the surface density states caused by ions.

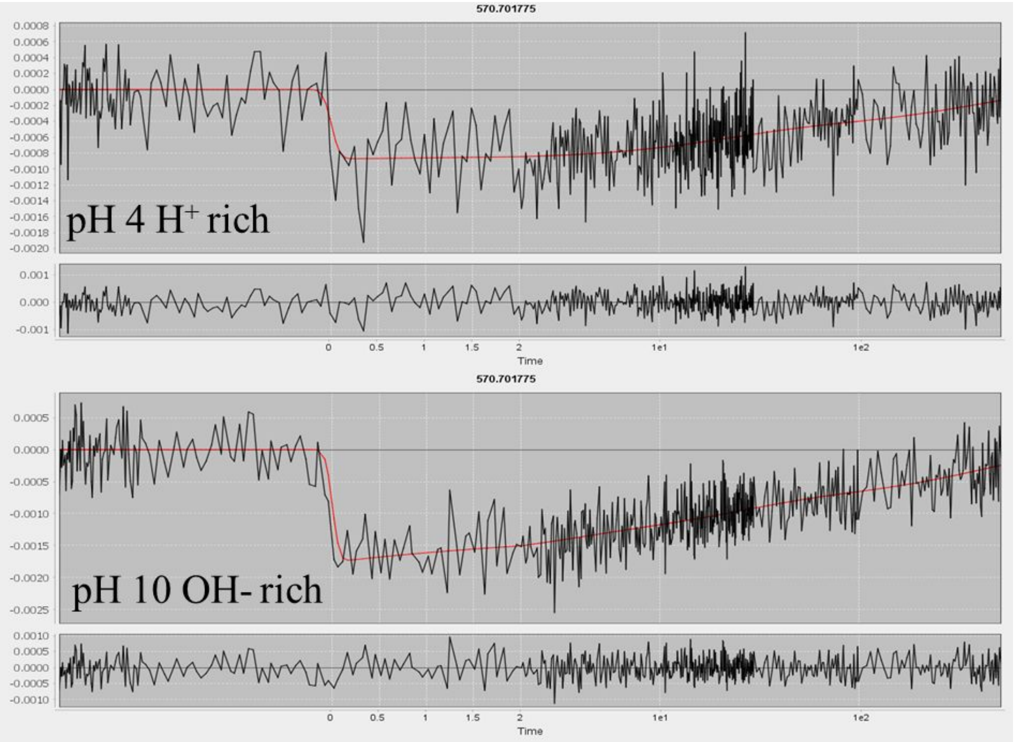

Figure S11. Transient absorption kinetic traces at 570 nm after excitation at 500 nm of same NiO/P1 sample in the solution with different pH.

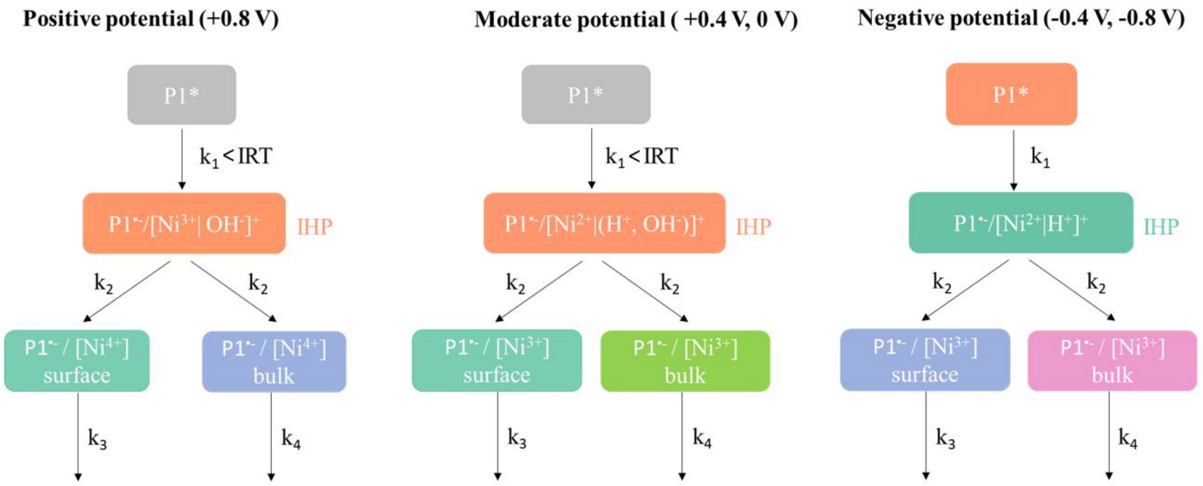

Figure S12. Photophysical model used to describe the femtosecond transient absorption data, IRT = instrumental response time (100-150 fs), IHP = inner Helmholtz plane. The colors of the boxes

correspond to the color of the species associated spectra shown in Figure S10. The grey boxes have not been included, as they decay within the IRT. The obtained time constants are presented in Table S1.

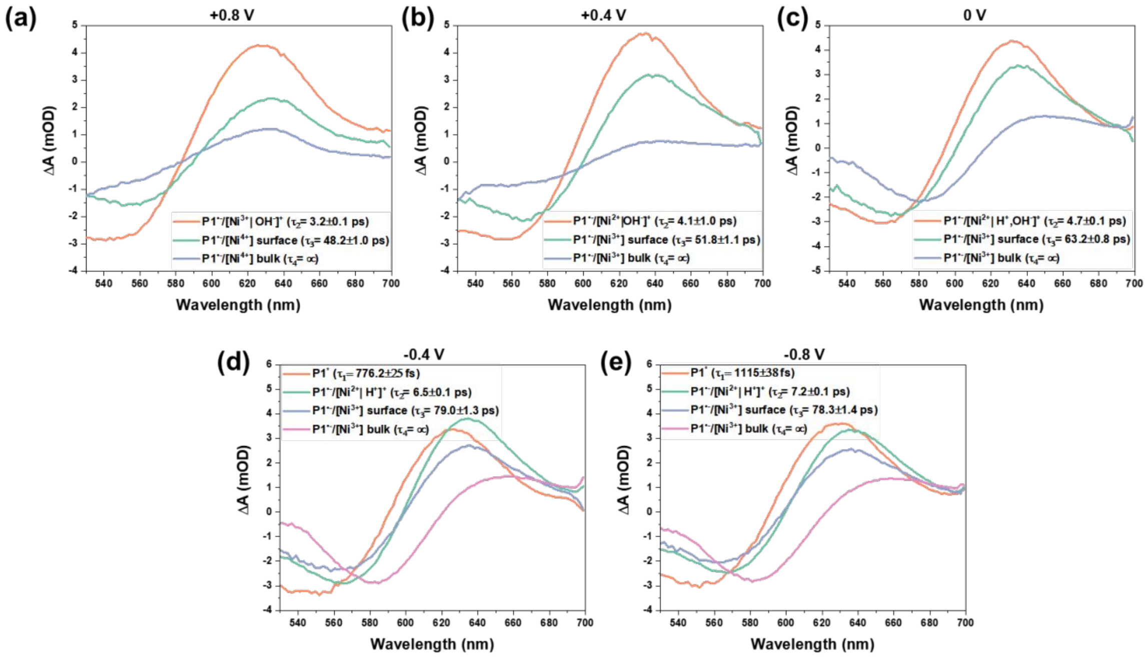

Figure S13. Species associated spectra obtained from target analysis of the TA data of NiO/P1 in PBS electrolyte (pH=7) under various external potentials vs. Ag/AgCl.

Table S1. Time constants from target analysis of NiO/P1 in PBS electrolyte (pH=7) under various external potentials vs. Ag/AgCl. IRT = instrumental response time (100-150 fs).

Potential (V)	τ_1 (fs)	τ_2 (ps)	τ_3 (ps)	τ_4 (ps)
+0.8	<IRT	3.2 ± 0.1	48.2 ± 1.0	∞
+0.4	<IRT	4.1 ± 0.1	51.8 ± 1.1	∞
0	<IRT	4.7 ± 0.1	63.2 ± 0.8	∞
-0.4	776.2 ± 25	6.5 ± 0.1	79.0 ± 1.3	∞
-0.8	1115 ± 38	7.2 ± 0.1	78.3± 1.4	∞

The transient absorption data and role of the applied bias potential are well described by the photophysical model shown in Figure S12, as apparent from the fits included as solid lines in Figures 3

and 4 obtained from target analysis using the open source program Glotaran.[11] A likely reason for the impact of the external bias potential observed is a strong dependency of the NiO surface termination on the working environment. Considering the dual role of surface OH^- we unraveled recently, accelerating both hole injection and charge recombination,[2] the dependency of the interfacial photodynamics on the external potential is likely due to a change in ions (H^+ or OH^-) in the inner Helmholtz plane (IHP). Figure S13 shows the species associated spectra from target analysis and Table S1 presents the derived time constants. At positive potentials (+0.8 V), photoinduced hole injection from P1* into the NiO (τ_1) is assumed to occur within the instrumental response time (IRT), promoted by the abundant surface adsorbed OH^-.[2] The early-time spectra are hence mainly due to $P1^{\bullet -}$ and the hole injected into the IHP. The hole likely localizes at a Ni^{3+} site, oxidizing it into a Ni^{4+} site[12] (τ_2) causing a red-shift in spectrum and finally decays either by surface or bulk charge recombination (τ_3 and τ_4). At moderate potential (+0.4 V, 0 V) the same model still applies, but now the holes injected by P1* are assumed to localize at Ni^{2+} sites instead of Ni^{3+} sites. At negative applied potentials (-0.4 V, -0.8 V) the quantity of surface OH^- promoting hole injection is likely very low, instead the NiO surface can be expected to be H^+ terminated. As a result, light-induced hole injection from P1* into the IHP is slowed down to >IRT (τ_1 = 776.2 ± 25 fs at -0.4 V and 1115 ± 38 fs at -0.8 V) and the early-time spectra are mainly due to P1*. Hole injection causes a red-shift in spectrum due to the decay of P1* and formation of $P1^{\bullet -}$. Holes injected into the NiO are again likely localized at Ni^{2+} sites, subsequently oxidizing them into Ni^{3+} sites (τ_2) causing a further red-shift in spectrum, and finally decaying by surface or bulk charge recombination (τ_3 and τ_4).

References

1. Cai, G.; Wang, X.; Cui, M.; Darmawan, P.; Wang, J.; Eh A. L.; Lee, P. S., Electrochromo-supercapacitor based on direct growth of NiO nanoparticles. *Nano Energy,* **2015**, *12,* 258-267.

2. Zhu, K.; Frehan, S. K.; Mul, G.; Huijser, A., Dual Role of Surface Hydroxyl Groups in the Photodynamics and Performance of NiO-Based Photocathodes. *J. Am. Chem. Soc.* **2022**, *144*, 11010-11018.

3. Zhu, K.; Frehan, S. K.; Jaros, A. M.; O'Neill, D. B.; Korterik, J. P.; Wenderich, K.; Mul, G.; Huijser, A., Unraveling the Mechanisms of Beneficial Cu-Doping of NiO-Based Photocathodes. *J. Phys. Chem. C*, **2021**, *125*, 16049-16058.

4. Qin, P.; Zhu, H. J.; Edvinsson, T.; Boschloo, G.; Hagfeldt, A.; Sun, L. C., Design of an organic chromophore for p-type dye-sensitized solar cells. *J. Am. Chem. Soc.* **2008**, *130*, 8570-8571.

5. Zhu, K.; Luo, W.; Zhu, G.; Wang, J.; Zhu, Y.; Zou, Z.; Huang, W., Interface-Engineered $Ni(OH)_2$/β-like FeOOH Electrocatalysts for Highly Efficient and Stable Oxygen Evolution Reaction. *Chem. Asian J.* **2017**, *12*, 2720-2726.

6. Corby, S.; Tecedor, M.-G.; Tengeler, S.; Steinert, C.; Moss, B.; Mesa, C. A.; Heiba, H. F.; Wilson, A. A.; Kaiser, B.; Jaegermann, W., Separating bulk and surface processes in NiOx electrocatalysts for water oxidation. *Sustain. Energ. Fuels* **2020**, *4*, 5024-5030.

7. Rao, R. R.; Corby, S.; Bucci, A.; García-Tecedor, M.; Mesa, C. A.; Rossmeisl, J.; Giménez, S.; Lloret-Fillol, J.; Stephens, I. E.; Durrant, J. R., Spectroelectrochemical Analysis of the Water Oxidation Mechanism on Doped Nickel Oxides. *J. Am. Chem. Soc.* **2022**, *144*, 7622-7633.

8. Zhang, Z.; Yates Jr, J. T., Band bending in semiconductors: chemical and physical consequences at surfaces and interfaces. *Chem. Rev.* **2012**, *112*, 5520-5551.

9. Meyer, G. J.; Lisensky, G. C.; Ellis, A. B., Evidence for adduct formation at the semiconductor-gas interface. Photoluminescent properties of cadmium selenide in the presence of amines. *J. Am. Chem. Soc.* **1988**, *110*, 4914-4918.

10. Wrede, S.; Cai, B.; Kumar, A.; Ott, S.; Tian, H., Lateral Electron and Hole Hopping between Dyes on Mesoporous ZrO2: Unexpected Influence of Solvents with a Low Dielectric Constant. *J. Am. Chem. Soc.* **2023**, *145*, 11472-11476.

11. Snellenburg, J. J.; Laptenok, S.; Seger, R.; Mullen, K. M.; van Stokkum, I. H., Glotaran: A Java-based graphical user interface for the R package TIMP. *J. Stat. Software* **2012,** *49*, 1-22.

12. D'Amario, L.; Föhlinger, J.; Boschloo, G.; Hammarström, L., Unveiling hole trapping and surface dynamics of NiO nanoparticles. *Chem. Sci.* **2018,** *9*, 223-230.
